# TRP Channels in Human Prostate

**DOI:** 10.1100/tsw.2010.149

**Published:** 2010-08-17

**Authors:** Carl Van Haute, Dirk De Ridder, Bernd Nilius

**Affiliations:** ^1^ Department of Molecular Cell Biology, Laboratory of Ion Channel Research, Campus Gasthuisberg, KULeuven, Belgium; ^2^ Department of Surgery, Laboratory of Experimental Urology, Campus Gasthuisberg, KULeuven, Belgium

**Keywords:** prostate, prostate cancer, prostate diseases, transient receptor potential channels, TRP

## Abstract

This review gives an overview of morphological and functional characteristics in the human prostate. It will focus on the current knowledge about transient receptor potential (TRP) channels expressed in the human prostate, and their putative role in normal physiology and prostate carcinogenesis. Controversial data regarding the expression pattern and the potential impact of TRP channels in prostate function, and their involvement in prostate cancer and other prostate diseases, will be discussed.

## INTRODUCTION

The prostate, a mainly exocrine gland that is conserved in all male mammals, is not essential for the survival of the individual, but it plays an essential role in the reproduction of the species. Prostate excision abolishes fertilization via natural ways, whereas artificial insemination remains achievable, suggesting a crucial role in natural male fertility[1].

### Morphology of the Prostate

As well as an exocrine gland, the prostate is also a fibromuscular stroma organ that surrounds the urethra just below the bladder. Together with the seminal vesicles, the prostate produces the gross of human seminal fluid, or ejaculate, released during the emission phase of copulation. The secreted prostate fluid contains citric acid, lipids, proteolytic enzymes (like prostate-specific antigen [PSA]), zinc (with 50 mg/100 g dry weight, the prostate has the highest concentration of zinc of any organ), and acid phosphatase, which are essential for sperm motility and viability, needed to achieve natural fertilization of the oocyte[1,2,3].

Morphologically, the prostate is a heterogeneous organ, made up of glandular and nonglandular parts, which are tightly fused within a common capsule. It contains three major glandular regions: the central zone, which is relatively resistant to disease; the peripheral zone, known as the most susceptible region for inflammation and carcinogenesis; and the transition zone, where benign prostatic hyperplasia (BPH)

occurs. The nonglandular region is mainly composed of the anterior fibromuscular region, constituting 30% of the prostate mass[4,5] (Fig. 1).

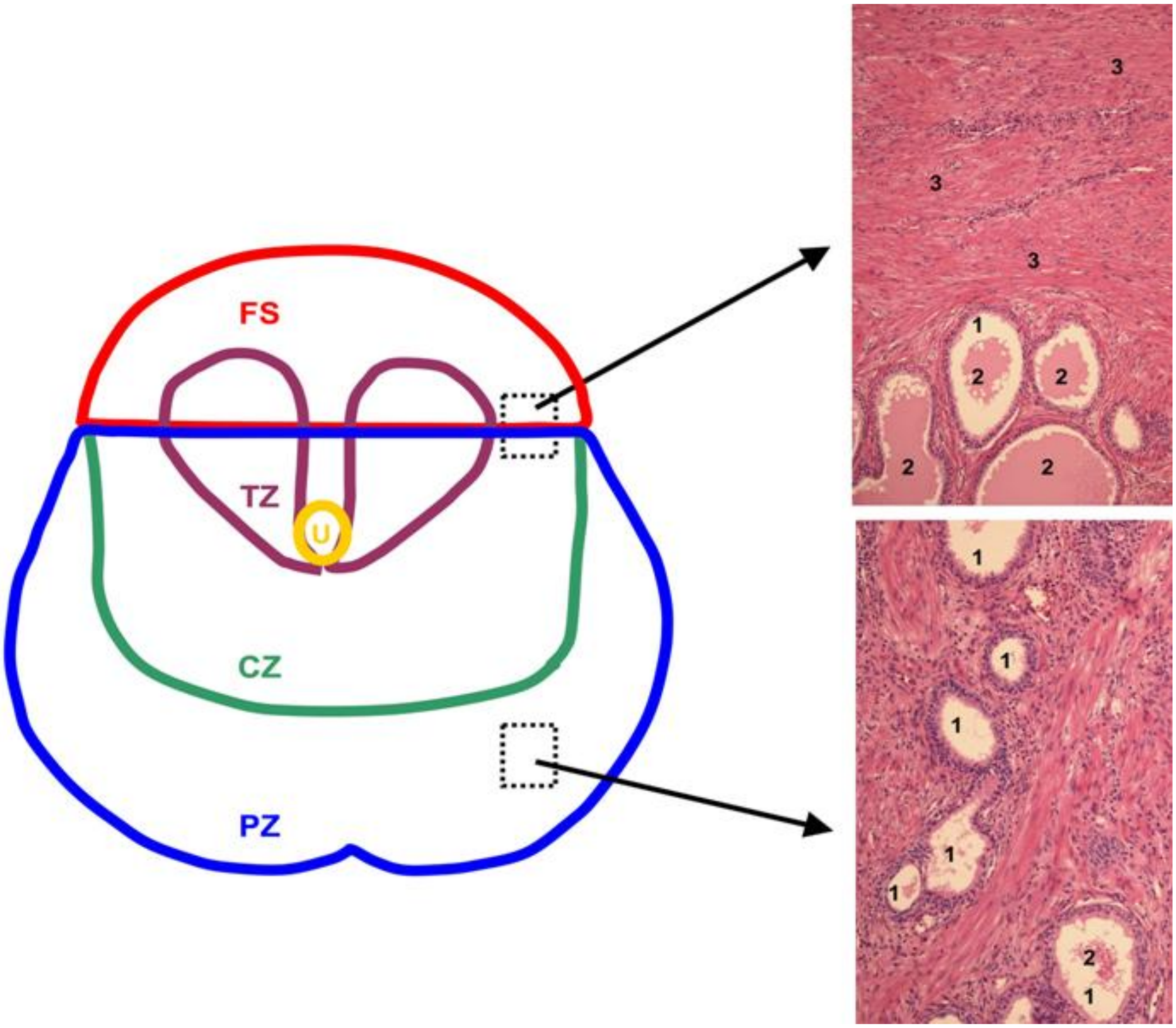

**FIGURE 1.** Zones in the human prostate (FS = fibromuscular stroma, TZ = transition zone, U = urethra, CZ = central zone, PZ = peripheral zone) with two 100× magnifications showing glandular and stromal parts of human prostate (1 = secretory parts of prostate gland, 2 = prostate secretions in the secretory parts, 3 = stroma, mainly consisting of smooth muscle cells and connective tissue). Stromal tissue comprises 30% of the prostate.

The glandular parts consist of pluripotent stem cells; basal epithelial cells residing in the basal compartment of the epithelial layer and expressing CK5 and CK14; transit amplifying cells in the basal layer, which form an intermediate cell type between undifferentiated stem cells and fully differentiated secretory cells; neuroendocrine cells that regulate growth, differentiation, and the secretory activity of the epithelium via paracrine secretion of hormones; and terminally differentiated luminal secretory cells, clustered in acini and morphologically characterized by abundant secretory granules and enzymes[6,7]. Acini drain into a system of branching epithelial ducts and tubules that end up directly in the urethra.

The nonglandular part or stroma is composed of smooth muscle cells, fibroblasts, connective tissue, and blood vessels. This part is highly innervated, mainly by adrenergic efferents.

## Innervation of the Prostate

The majority of the acini contains a dense subepithelial plexus of nerves[8] and several autonomic ganglia are clustered at the capsule surface[5]. In the past, it was generally believed that the prostate was exclusively innervated by sympathetic efferents; however, ultrastructural imaging studies on the human prostate also showed the presence of cholinergic nerve fibers, mainly associated with the glandular epithelium[9]. Some studies even suggested that the density of cholinergic nerve fibers exceeded that of adrenergic nerve fibers in the overall prostate[10]. Adrenergic receptors consist mainly of $\alpha_1$-adrenoceptors ($\alpha_{1A} > \alpha_{1D}$), primarily localized to the prostate fibromuscular stroma, and $\alpha_2$-adrenoceptors ($\alpha_{2A}$, $\alpha_{2B}$, $\alpha_{2C}$), mainly associated with blood vessels[2,11]. Muscarinic receptors in the prostate mainly belong to the M1 subtype and are expressed on epithelial cells at the protein level; a smaller population of M2 receptors is found on the stromal cells[12]. Parasympathetic outflow to the prostate stimulates the secretion of prostate fluids, whereas sympathetic efferents evoke expulsion of prostate fluids during emission. The significance of the nonadrenergic-noncholinergic (NANC) nerves, also present in the prostate, is still unclear[13].

## Prostate Cancer

Prostate cancer (PCa) is the most commonly diagnosed noncutaneous cancer in men, and the second and third most common cause of cancer-related death in North America and Europe, respectively[14,15]. Moreover, the number of afflicted men is increasing rapidly as the population of males over the age of 50 continues to grow.

In clinical practice, PCa is usually diagnosed by an abnormal digital rectal examination or by finding elevated PSA levels in the blood. The diagnosis is then confirmed by transrectal ultrasound (TRUS)–guided biopsies, which are pathologically examined and scored according to the Gleason scoring system. The Gleason score is the most frequently used grading system for PCa, used to evaluate the differentiation grade of the tumor; it is based on scoring the two most predominant glandular differentiation patterns in the prostate sample[16].

Although organ-confined PCa is curable with radical prostatectomy or radiotherapy, there are only limited treatment options for metastasized disease (hormonal treatment, chemotherapy). Huggins and Hodges described that surgical orchidectomy leading to androgen ablation was an effective and very reproducible treatment for metastatic PCa[17], a discovery for which they were awarded the Nobel Prize in 1966. This treatment is founded on the principle that prostate epithelial cells depend on androgens for their survival, as castration in a male rat leads to a loss of up to 90% of the total epithelial cells[18]. Unfortunately, androgen deprivation, either by surgical or chemical castration, or by administration of androgen receptor inhibitors, is unable to destroy all tumoral cells and over a period of time, growth of androgen-independent prostate tumor cells will lead to tumor progression, regardless of the hormonal status of the patient. Hence, PCa progresses and recurs during hormone therapy to a lethally resistant phenotype[19].

## Transient Receptor Potential Channels

The **t**ransient **r**eceptor **p**otential (**TRP**) channel superfamily consists in mammalian species of 28 cation-permeable channels that are ubiquitously expressed and share a high degree of structural homology, i.e., they form tetramers in which each TRP channel subunit consists of six putative transmembrane segments, a putative pore-forming loop between S5 and S6, and intracellularly located amino- and carboxytermini[20,21]. Based on homology criteria, mammalian TRP channels can be divided into six subfamilies: TRP**C** (**c**anonical or **c**lassical), TRP**V** (**v**anilloid), TRP**M** (**m**elastatin), TRP**ML** (**m**uco**l**ipin), TRP**A** (**a**nkyrin-like), and TRP**P** (**p**olycystin).

A typical feature of these TRP channels is their ability to be activated by a wide range of chemical and mechanical stimuli. As such, they can be envisioned as the polymodal molecular sensors of the cell. Most, but not all, of the TRP channels function as $Ca^{2+}$ pathways, cause cell depolarization, and also form intracellular pathways for $Ca^{2+}$ release from various intracellular stores, such as the endo- and sarcoplasmic reticulum, lysosomes, and endosomes[22]. Beyond their sensory functions, they are broadly involved in diverse homeostatic functions. It is not surprising, therefore, that dysfunctions of these TRP channels are involved in the pathogenesis of several diseases[20,21,23,24].

Many TRP channels have so far been described in the genitourinary tract[25,26] and more specifically in the prostate, where they are suggested to play a role in normal prostate physiology and prostate diseases, most importantly in prostate carcinogenesis. This paper will review the current scientific evidence about the expression of TRP channels in human prostate and their possible role in prostate diseases.

## TRPM8

## Expression Pattern of TRPM8

TRPM8 was cloned in 2001 as a novel prostate-specific gene by screening a prostate cDNA library[27]. One year later, it was shown that TRPM8 encodes for a cold- and menthol-sensitive ion channel in trigeminal ganglion and dorsal root ganglion neurons (TGN and DRG)[28,29,30].

Using *in situ* hybridization on paraffin-embedded sections, it was shown that TRPM8 mRNA was expressed solely in the epithelial cells of the prostate, but not in the vascular smooth muscle cells and endothelium. Moreover, the mRNA levels in BPH and PCa appeared to be higher than in the normal prostate[27].

TRPM8 was shown to be expressed at the protein level via immunohistochemistry[31,32] and Western blot experiments[32], both in the apical epithelial cells and in the smooth muscle cells of the human prostate[31,32].

Several groups have studied the expression of TRPM8 in different PCa cell lines. In primary cultures of prostate epithelial cells, the density of the TRPM8 membrane current was increased in cancerous compared to normal cells. These currents exhibited classical cold/menthol receptor-like responses (strong outward rectification and close to 0 mV reversal potential)[32]. Moreover, RT-PCR analysis of these cells revealed an up-regulation of TRPM8 in PCa-derived cells[32].

Also in tumoral cell lines, such as LNCaP (“lymph node carcinoma of the prostate”, a widely used cell line derived from a supraclavicular lymph node metastasis expressing the androgen receptor [AR][33]), TRPM8 was detected by RT-PCR[27]. Zhang and Barritt also suggested a functional role for TRPM8 in LNCaP cells, since temperatures below 28°C or application of 100 μM menthol, which is sufficient for TRPM8 activation, led to an increase in $[Ca^{2+}]_{cyt}$[34,35]. In contrast, Mahieu et al. provided convincing evidence that menthol-induced $Ca^{2+}$ release from intracellular stores in LNCaP cells was not mediated by TRPM8[36]. Moreover, other authors also rejected a functional role for TRPM8 in LNCaP cells, since the application of WS-12, icillin, or menthol was without any effect in $Ca^{2+}$ imaging experiments[37,38].

Regarding the localization of TRPM8 in LNCaP, Thebault et al. reported that TRPM8 was almost exclusively expressed in the endoplasmic reticulum ($_{ER}$TRPM8)[39], whereas Mahieu et al. reported a mainly plasmamembrane localization of TRPM8 ($_{PM}$TRPM8)[36].

## Androgen Regulation of TRPM8

Henshall et al. suggested that TRPM8 was androgen regulated, as there was a decrease of TRPM8 mRNA expression in a xenograft mouse PCa model after castration[40]. Moreover, TRPM8 mRNA levels in LNCaP cells decreased significantly on withdrawal of androgens[34] or by treatment of the cells with AR

antagonists[31]. It is doubtful, however, that the AR is essential for TRPM8 expression, since in PC-3 cells, which lack the AR, TRPM8 is expressed[34]. The latter has been contested by other authors[32].

Further, Bidaux et al. reported that TRPM8 expression requires a functional AR. Transfection of the AR into PNT1A cells, which lack the expression of the AR in normal physiological conditions, induced the appearance of TRPM8 that could be reversed by incubation of siRNA-AR[31]. Using single-cell RT-PCR and immunohistochemistry, they showed that TRPM8 is mainly located in the apical epithelial secretory cells that express CK8 and CK18, and not in the basal epithelial cells expressing CK5 and CK14[31]. Primary cultures of prostate epithelial cells expressed the AR, TRPM8, CK8, and CK18 after 12 days, but after 20 days, the cultured cells displayed a more basal epithelial phenotype, expressing CK5 and CK14, but not the AR and TRPM8[32]. Moreover, it seems that the AR regulates the membranic translocation of TRPM8, since TRPM8 resides in the ER in the absence of the AR, and only appears in the plasmamembrane when the AR is expressed. The authors postulated the hypothesis of a shift of $_{PM}$TRPM8 in normal apical fully differentiated epithelial cells to $_{ER}$TRPM8 in a metastatic PCa cell during prostate carcinogenesis[32].

## Clinical Relevance of TRPM8

So far, only a few studies have been conducted on the clinical relevance of altered TRPM8 expression in PCa.

PSA (or human kallikrein 3), a glycoprotein that acts as a serine protease, is the most frequently used PCa biomarker in the blood that is used in the detection and follow-up of PCa. It is, however, not a perfect tumor marker, lacking both the sensitivity and the specificity to detect PCa. A recently published randomized controlled trial concluded that the rate of death of PCa was reduced by 20% due to PSA screening, but this was associated with a high risk of false-positive diagnoses and treatments for indolent cancers[41]. Thus, there is need for a more appropriate biomarker.

Several studies using quantitative RT-PCR revealed a significantly increased expression of TRPM8 mRNA in malignant prostate samples in comparison to nonmalignant tissue, suggesting that the level of TRPM8 in biopsy specimens could be used in the diagnosis of PCa[42,43]. This elevation seemed to be statistically significant, unlike the relative transcript-level elevation of PSA mRNA[42]. However, no clear correlation of TRPM8 expression with the pathological grade of PCa could be found[43]. Moreover, Henshall et al. showed a strong correlation between the level of TRPM8 mRNA expression and disease relapse after radical prostatectomy, as loss of TRPM8 was associated with a significantly shorter time to PSA relapse-free survival[40].

Moreover, TRPM8 was even suggested as a possible target structure for immunotherapy of prostate tumors by generating cytotoxic T lymphocytes that could lyse TRPM8-expressing LNCaP cells[43].

## Role of TRPM8 in Prostate

In prostate carcinogenesis, the role of TRPM8 remains unclear, although different roles have been suggested.

It has been suggested that TRPM8 plays a role in PCa cell proliferation since siRNA-induced silencing of TRPM8 in LNCaP cells led to an increased number of cells undergoing apoptosis. Similarly, application of 10 μM capsazepine, a TRPM8 antagonist, decreased cell viability of the LNCaP cells, indicating that TRPM8 is required for LNCaP survival[34]. In primary cultures of prostate cells, the application of 10 μM geraniol, a known TRPM8 agonist, led to an increased cell proliferation[44].

In addition to a possible role in PCa cell proliferation, it has also been suggested that TRPM8 is a $Ca^{2+}$ release channel in the ER. In patch clamp experiments, Thebault et al. showed that the application of 100 μM menthol on LNCaP cells did not exhibit a classical TRPM8-mediated current, but showed the characteristics (inward rectification and divalent cation selectivity) of a current mediated through a storeoperated channel (SOC). They reported a role for TRPM8 in LNCaP as a cold/menthol-sensitive ER $Ca^{2+}$ release channel, and that the $I_{SOC}$ activation was secondary to menthol-mediated ER store depletion[39], as was suggested by Zhang and Barritt[34].

Other possible roles for TRPM8 have also been suggested. Since TRPM8 is colocalized with CK18 in primary epithelial prostate cultures, a role for TRPM8 as an epithelial phenotype stabilizer has been proposed[32]. TRPM8 could also act as an oncogene, since it is up-regulated in prostate adenocarcinoma, but also in other neoplastic lesions, such as colon carcinoma, melanoma, and breast and lung adenocarcinoma[27]. Lastly, Zhang and Barritt suggested a role for TRPM8 in ion and protein secretions in prostate epithelial cells[34].

## TRPV6

### Expression Pattern of TRPV6

TRPV6, an epithelium TRP channel highly selective for $Ca^{2+}$ in organs that reabsorb $Ca^{2+}$, was originally cloned from rat duodenum as a $Ca^{2+}$ transport protein[45]. Northern blot analysis showed strong expression of TRPV6 transcripts in the prostate[46]. In 2001, using Northern blot and *in situ* hybridization, Wissenbach et al. described that TRPV6 was present in PCa tissue specimens and in lymph node metastasis, but not in BPH or in normal prostate. The most elevated levels of TRPV6 mRNA were found in high-grade, locally advanced (pT3a/b) prostate tumors, whereas no TRPV6 mRNA was detectable in low-grade PCa, suggesting that TRPV6 could be a promising prostate tumor marker[47]. Two allelic variants of TRPV6 have been described, TRPV6a and TRPV6b, differing in five base pairs, but not encoding for ion channels with different properties. The onset of PCa seemed to be independent of the TRPV6 genotype[48].

In 2001, Peng et al.[51] confirmed these results via *in situ* hybridization experiments, but claimed that TRPV6 was also expressed in normal epithelial cells, BPH tissue, and LNCaP cells.

TRPV6 was also detected at the protein level in immunohistochemistry experiments and its up-regulation was also demonstrated in other malignancies in the breast, thyroid, colon, and ovary[49].

Interestingly, TRPV6 mRNA expression correlated significantly with the Gleason score and the pathological stage (TRPV6 was absent in normal prostate, BPH, and pT1a/b lesions, but appeared in higher pathological stages)[50].

### Androgen Regulation of TRPV6

Peng et al. suggested in 2001 that TRPV6 expression was androgen controlled, showing that the administration of AR antagonists to LNCaP cells resulted in a twofold increase of TRPV6 mRNA levels, whereas adding dihydrotestosterone (DHT) decreased TRPV6 levels[51]. In contrast, TRPV6 mRNA expression studies revealed decreased TRPV6 expression levels in androgen-deprived human prostates[50]. Other authors found that the application of AR antagonists or DHT had no significant effects on TRPV6 expression in LNCaP cells at all[52]. siRNA knockdown of the AR, however, induced a significant decrease of TRPV6 expression. Moreover, it was suggested that TRPV6 was regulated by the AR in a ligand-independent manner and that the AR constituted an essential cofactor of TRPV6 gene transcription in LNCaP cells[52].

### TRPV6 and $K_{Ca}3.1$

Cell hyperpolarization will always increase the driving force for $Ca^{2+}$ entry via $Ca^{2+}$-permeable ion channels, such as TRPV6. $Ca^{2+}$ entry via these channels depends on coactivation of the intermediateconductance, calcium-activated, potassium channels ($IK_{Ca}$ or according to the IUPHAR nomenclature $K_{Ca}3.1$ or SK41)[53], which are expressed in LNCaP cells as well as in primary prostate epithelial cultures. Moreover, $K_{Ca}3.1$ seemed to be preferentially expressed in PCa tissue, leading to hyperpolarization of the plasma membrane, after which TRPV6 is opened and $Ca^{2+}$ influx occurs. siRNA knockdown of $K_{Ca}3.1$ and blocking of $K_{Ca}3.1$ led to a decreased cell proliferation in LNCaP[54].

## Role of TRPV6 in Prostate

Authors suggested a role for TRPV6 in cell proliferation. TRPV6 increased the proliferation rate of HEK cells in a $Ca^{2+}$-dependent manner. As TRPV6 slightly enhanced global resting $[Ca^{2+}]_{IC}$, these small changes could indeed increase proliferation rate. This suggests a causal relationship between PCa progression and TRPV6 expression[55].

Lehen'kyi et al. showed that silencing assays of TRPV6 in LNCaP led to a decreased number of viable cells. They suggested a role for TRPV6 in LNCaP proliferation by mediating $Ca^{2+}$ entry, which is followed by the activation of $Ca^{2+}$-dependent NFAT ("nuclear factor of activated T cells", a nuclear transcription factor) signaling pathways. As such, TRPV6 increased cell survival and induced apoptosis resistance[52].

## TRPC

The TRPC subfamily consists of mammalian TRP channels that are most closely related to *Drosophila* TRP. TRPC channels can be considered as channels activated subsequent to stimulation of receptors that activate different isoforms of phospholipase C[20].

## Expression Pattern of TRPC Channels

The first TRPC channel to be described in the prostate was TRPC3. Using Northern blot analysis, the expression of this gene was described in the normal prostate[56]. On the other hand, a more extensive quantitative TRP expression study in human prostate samples revealed the abundant expression of TRPC1, TRPC4, and TRPC6, whereas TRPC3, TRPC5, and TRPC7 were hardly detected[57].

In addition to normal prostate, immunohistochemistry revealed expression of TRPC6 in BPH and, more importantly, a significant overexpression in PCa specimens. Higher pathological stages of PCa tended to have increased TRPC6 expression, but these differences were not statistically significant among pT2, pT3, and pT4 PCa[58].

Human primary prostate epithelial cell cultures expressed TRPC1A, a TRPC1 splice variant, TRPC3, TRPC4 (and the splice variant TRPC4β), and TRPC6 (and the splice variant TRPC6γ) at the mRNA level[59]. In LNCaP, the presence of TRPC1, TRPC3, TRPC5, and TRPC7 was detected; TRPC6, however, seemed not to be present[60].

## Role of TRPC Channels in Prostate

The functional role of TRPC channels in the prostate has been investigated in human primary prostate epithelial cell cultures, using antisense assays of TRPC1, TRPC3, TRPC4, and TRPC6. It was postulated that TRPC1 and TRPC4 were exclusively involved in ATP-stimulated, store-dependent $Ca^{2+}$ entry (SOCE), whereas TRPC6 was the diacylglycerol-gated, channel mediating $\alpha_1$-AR ($\alpha_1$-adrenergic receptor) agonist–stimulated $Ca^{2+}$ influx (store independent). Moreover, treatment of the cultures with $\alpha_1$-AR agonists enhanced cell proliferation, in contrast to ATP, which had an inhibitory effect. Therefore, the authors concluded that TRPC6 is a crucial mediator of the proliferative effects of $\alpha_1$-AR agonists. TRPC1 and TRPC4, on the other hand, are the major contributors of SOC activation in response to ATP[59].

In LNCaP, TRPC1 and TRPC3 were overexpressed after prolonged intracellular $Ca^{2+}$ store depletion due to the decreased levels of $[Ca^{2+}]_{CYT}$. LNCaP cells overexpressing TRPC1 and TRPC3 showed an increased $[Ca^{2+}]_{IC}$ response to α-adrenergic stimulation, but SOCE entry remained unaffected. Thus, expression of TRPC1 and TRPC3 is not sufficient for SOC formation[60].

## TRPM2

Recently, the presence of TRPM2 has been demonstrated in laser microdissected, tumoral epithelial, human prostate cells using quantitative RT-PCR. The analysis showed a high expression of TRPM2 transcripts in 75% of the malignant epithelial cells in comparison to the matched microdissected benign cells of the surgical specimens. In addition, TRPM2 RNA was detected in LNCaP and PC-3 cells. In PC-3 cells, TRPM2 was not only expressed in the plasmamembrane and the cytosol, but also in the nucleus, in contrast with benign cell lines where nuclear expression was absent[61]. Importantly, TRPM2 is also expressed in lysosomes and may act as a lysosomal $Ca^{2+}$ release channel[62]. Furthermore, siRNA knockdown of TRPM2 inhibited cell growth in PC-3 cells, but not in benign prostate cells, suggesting that TRPM2 is essential for PCa cell proliferation[61].

## TRPM4

TRPM4, a nonselective cation channel activated by intracellular $Ca^{2+}$, was cloned in 2001 after screening a brain, placenta, and testis cDNA library. Via Northern blot analysis, TRPM4 was also detected in prostate tissue[63]. Later it turned out that this clone was a short splice variant of the full-length TRPM4. The full-length TRPM4 was subsequently called TRPM4b, while the short splice variant was baptized TRPM4a. The presence of TRPM4b transcripts in prostate (adenocarcinoma) was confirmed in two different studies[64,65].

## TRPV1

TRPV1, a heat-activated, nonselective cation channel, was originally identified as the receptor for capsaicin, the pungent ingredient in hot chilli peppers. It is one of the most polymodal TRP channels, being activated, among others, by heat, voltage, protons, and exogenous (capsaicin, piperidine) and endogenous vanilloids (anandamide and 2-arachidonoylglycerol [2-AG])[66], and is mainly expressed in sensory neurons[20,67].

TRPV1 expression has been described at the mRNA[68] and protein[69] levels in the human prostate and in LNCaP[69]. TRPV1 expression is up-regulated in high-grade PCa, in comparison to normal prostate[70]. Moreover, using a competitive resiniferatoxin (RTX) binding assay (in BPH cells and LNCaP cells) and $Ca^{2+}$ imaging experiments (in LNCaP cells), authors reported that TRPV1 was functionally active in BPH and LNCaP[69]. Regarding the localization, immunohistochemistry showed a predominant expression of TRPV1 in nerves throughout the prostate[71].

It was reported that capsaicin inhibits the growth of PC-3 cells with an $IC_{50}$ of 20 μM[72]. However, these apoptotic effects were not mediated by TRPV1, but resulted from a direct inhibiting effect of vanilloids on Coenzyme Q, which promotes reactive oxygen species production resulting in apoptosis, and an activation of caspase 3. In line with this hypothesis, capsazepine, a TRPV1 antagonist, could not block capsaicin-induced inhibition of PC-3 cell growth[72]. An indirect apoptosis pathway mediating TRPV1 was also suggested through the increase of $[Ca^{2+}]_{IC}$ upon capsaicin application, leading to an activation of $Ca^{2+}$-dependent enzymes, such as endonucleases, proteases, and transglutaminases, resulting in DNA injury, cytoskeleton damage, and protein alteration, respectively[73].

Thus a clear role for TRPV1 in prostate cell proliferation has not been established.

In addition to its putative role in prostate cell proliferation, a role for TRPV1 has also been suggested in chronic prostatitis[71].

## TRPV2

TRPV2 was shown to be present in the prostate via immunohistochemistry experiments[74]. These channels might be activated by mechanical forces, by cannabinoids ($\Delta^9$-tetrahydrocannabinol, cannabidiol), heat, and by a plethora of chemical compounds, such as probenecide and 2-aminoethoxydiphenyl borate (2-APB)[75,76].

Recently, it was shown via quantitative RT-PCR that TRPV2 expression was 12 times higher in metastatic PCa samples (originating from the bone) than in localized PCa samples. TRPV2 was not expressed in LNCaP cells[77].

The authors further postulated that TRPV2 is *de novo* expressed in PCa tumor progression to a castration-resistant phenotype. Moreover, mice bearing xenografted PC-3 cell-provoked tumors were treated with siTRPV2. The weight of the tumors in mice treated with siTRPV2 was significantly smaller than in mice treated with siRNA control, an effect that contributed to the suppression of the migration of the cells. *In vitro*, TRPV2 did not exhibit effects on cell proliferation[77].

## TRPA1

TRPA1, mainly detected in DRG and TGN, and activated among others by noxious cold[78], allyl isothiocyanate[79] and bradykinin[80], plays a central role in nociception.

TRPA1 was detected at the mRNA level in the prostate. In immunohistochemistry experiments, it was postulated that TRPA1 resided in the prostate epithelial cells without a difference in expression between normal and BPH prostate[81]. Furthermore, TRPA1 is expressed on cannabinoid receptor (CB) 1- and CB2-positive nerve fibers in the stroma and the epithelium, and activation of these TRPA1-expressing fibers induced relaxation of prostate smooth muscle. In BPH, the immunoreactivity of TRPA1, CB1, and CB2 was significantly reduced[82].

Since TRPA1[82] and TRPV1[71], which are both important actors of nociception, are expressed along nerve fibers in prostate tissue, these channels can play a role in the pathogenesis of nonbacterial prostatitis and prostatodynia, and they might be considered as a new pharmacological target.

## OTHER TRPS

In addition to the TRPs described above, several studies suggested the expression of TRPM5[83,84], TRPM6[84], TRPM7[84], TRPV4[85], and TRPV5[86], although the evidence for this is still very limited.

## DISCUSSION

Although PCa is the most diagnosed nonskin cancer in men, there is a major lack of predictive prognostic biomarkers that can distinguish prospectively between aggressive and indolent disease. Although some factors, such as high Gleason score or short PSA doubling time, predict a worse prognosis, some patients will progress even with apparently low-risk disease.

Several TRP channels have been described in the prostate at the RNA level, the protein level, or the functional level using prostate material of diverse nature: freshly frozen human prostate tissue, paraffin-embedded tissue, LNCaP cells, PC-3 cells, and primary epithelial prostate cells. In addition, several of these channels have been identified as possible prognostic biomarkers of PCa.

Importantly, the results described above should be interpreted with care, taking into account that PCa research lacks a well-validated model.

LNCaP cells are widely used as an androgen-dependent, PCa epithelial cell model. However, this cell line was established from a metastatic, supraclavicular, lymph node lesion of a patient with hormone-refractory prostate adenocarcinoma. These LNCaP cells bear a mutation in the AR gene, encoding a promiscuous AR that can bind to other steroids than androgens. As such, it is questionable whether these cells are truly androgen dependent[87].

The role and the functional expression of TRPM8 in LNCaP cells are still a matter of debate; according to some authors, TRPM8 is functionally expressed [34], whereas other authors concluded that there is no functional role at all for TRPM8 in LNCaP cells[36,37,38].

Another controversial issue in LNCaP cells is the androgen regulation of TRPV6. Peng et al. showed that there was a twofold increase of TRPV6 mRNA levels upon application of 1 μM bicalutamide[51], an AR antagonist, whereas Lehen'kyi et al. reported that 10 μM bicalutamide had no significant effect on TRPV6 mRNA[52].

Therefore, the general impact of all TRPM8 and TRPV6 experiments performed in LNCaP cells on the development and treatment of PCa should be considered with caution given the diverse results, the doubtful prostate and epithelial nature of these cells, and their contested androgenic status. In addition, in PC-3 cells, another widely used cell line in PCa research, data about TRPM8 mRNA expression are contradictory[32,34].

Similarly, controversial data exist regarding the expression of TRPC channels in the different PCa models; TRPC3, TRPC5, and TRPC7 are expressed in LNCaP cells[60], while these channels have a very low expression level in human prostate specimens[57]. In contrast, TRPC6 is present in human prostate specimens at mRNA[57] and protein[58] levels, but not in LNCaP[60].

These conflicting data emphasize the definite need for better PCa models.

A more appropriate model for PCa research seems to be the use of primary epithelial cell cultures of normal and cancerous prostate tissue[32,59]. Unfortunately, due to the difficulty and labor intensiveness of this technique, primary cultures are not yet widely used and little is known about the epithelial characteristics of these cells.

The majority of all mentioned TRP channels have been described in “human prostate tissue”. As discussed before, the prostate is a mixture of a wide variety of cells. The studies reporting TRP channel expression nearly never distinguish between epithelial and stromal cells.

TRPM8, TRPV1, and TRPA1 are TRP channels with clearly defined roles in the sensory neuronal system[20]. These channels have been described in random prostate tissue[27,68,81], but it is unclear whether these channels are expressed in epithelial cells or in neuronal cells. The presence of a sensory nervous system in the prostate has long been ignored in urological literature and was demonstrated by McVary et al. in 1998[13]. By injecting a tracer in the ventral prostate of a rat for retrograde labeling of the afferent nerves, they showed that a sensory innervation was present in prostate tissue. The majority of afferent nerve fibers innervating the rat prostate projected to L5 and L6 DRG[13]. Therefore, it is possible that TRPM8, TRPV1, and TRPA1 expression in random human prostate tissue originates from afferent neurons innervating the prostate, rather than from the epithelial cells.

TRP expression studies are encountered with a lack of well-characterized and specific antibodies[88]. In the PCa literature, several studies concluded that TRPM8 was expressed in apical epithelial cells, solely based on immunohistochemistry experiments using a polyclonal TRPM8 antibody without a clear positive or negative control[31]. Thus, the antibody could have reacted nonspecifically.

The up-regulation of TRPM8[27] and TRPV6[47] in PCa tissue was demonstrated using *in situ* hybridization studies, which is not really a quantitative technique. One can wonder whether this technique was the most appropriate to conclude that TRPM8 and TRPV6 were up-regulated in PCa.

It was further demonstrated that TRPM8 was not only up-regulated in prostate adenocarcinoma, but also in other neoplastic lesions. Since TRPM8 is also up-regulated in other cancer types and since TRPM8 has a fairly established role in the sensory nervous system (for a review, see [30]), it is questionable whether TRPM8 really is a prostate-specific protein and thus can act as a new pharmacological target for PCa.

In summary, we can conclude that several TRP channels have been identified in the human prostate using non- or semi-quantitative methods, but no TRP channels have definite, clear roles in prostate physiology or carcinogenesis. The majority of the TRP expression studies in the human prostate have used random prostate tissue, whereas the prostate itself is an extremely heterogeneous organ. All TRP expression studies of the prostate should be cell-specific and should be read with care. Quantitative methods and functional data, if possible, are indispensable. There is a definite need for more appropriate prostate epithelial cell models, such as primary cultures of prostate epithelial cells, but the latter should be more thoroughly characterized.

**TABLE 1**
**Overview of TRP Channel Expression in (Random) Human Prostate, LNCaP Cells, and Human Primary Prostate Epithelial Cells**

| | LNCaP | Prostate Tissue | Human Primary Epithelial Cultures |
|---|---|---|---|
| TRPC1 | RNA[60] | RNA[57] | RNA[59] |
| TRPC3 | RNA[60] | RNA[56] | RNA[59] |
| TRPC4 | RNA[60] | RNA[57] | RNA[59] |
| TRPC5 | RNA[60] | | |
| TRPC6 | | RNA[57]; protein[58] | RNA[59] |
| TRPC7 | RNA[60] | | |
| TRPM2 | RNA[61] | RNA[61] | |
| TRPM4 | | RNA[63,64,65] | |
| TRPM5 | | RNA[83,84] | |
| TRPM6 | | RNA[84] | |
| TRPM7 | | RNA[84] | |
| TRPM8 | RNA[27,34]; protein[31,34,36] | RNA[27,68,84]; protein[31,32] | RNA[32]; protein[32] |
| TRPV1 | RNA[69]; protein[69] | RNA[68]; protein[69,70,71] | |
| TRPV2 | | RNA[77]; protein[74,77] | |
| TRPV4 | | RNA[85] | |
| TRPV5 | | RNA[86] | |
| TRPV6 | RNA[51,54,60]; protein[52] | RNA[46]; protein[49] | |
| TRPA1 | | RNA[81]; protein[81] | |

## ACKNOWLEDGMENTS

The authors would like to thank Wouter Everaerts for reading the manuscript and the helpful suggestions.

This work was supported by grants from the Belgian Federal Government (IUAP P6/28), the Research Foundation-Flanders (F.W.O.) (G.0565.07 and G.0686.09), the Astellas European Foundation award 2009, and the Research Council of the KU Leuven (GOA 2009/07 and EF/95/010). D.D.R. is a fundamental-clinical fellow of the F.W.O.

## ABBREVIATIONS USED IN TEXT

TRP, transient receptor potential; PSA, prostate-specific antigen; BPH, benign prostatic hyperplasia; NANC, nonadrenergic-noncholinergic; PCa, prostate cancer; TRUS, transrectal ultrasound; TGN, trigeminal ganglion neuron; DRG, dorsal root ganglion neuron; AR, androgen receptor; LNCaP, lymph node carcinoma of the prostate; ER, endoplasmic reticulum; PM, plasma membrane; CK, cytokeratin; SOC, store-operated channel; NFAT, nuclear factor of activated T cells; SOCE, store-dependent $Ca^{2+}$ entry; α1-AR, alpha 1 adrenergic receptor; RTX, resiniferatoxin; CB, cannabinoid receptor.
